# Supplementary figures and images for: High-content phenotyping reveals Golgi dynamics and their role in cell cycle regulation
Source: J Cell Biol. 2025 Nov 14;225(1):e202503083. doi: 10.1083/jcb.202503083 (PMC12617404; doi:10.1083/jcb.202503083)

## siJNK1

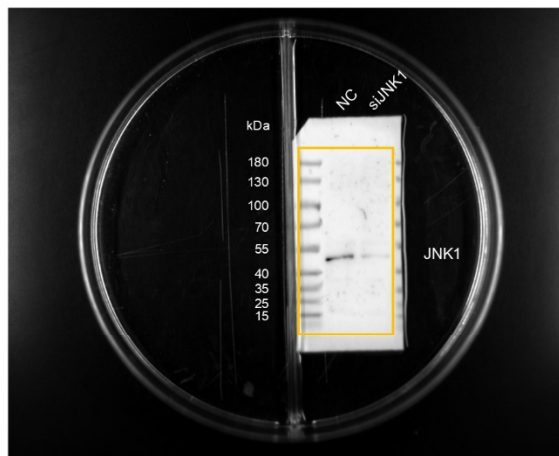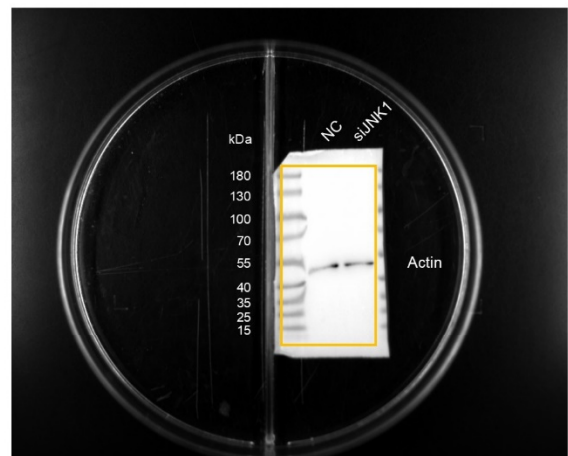

## siJNK2

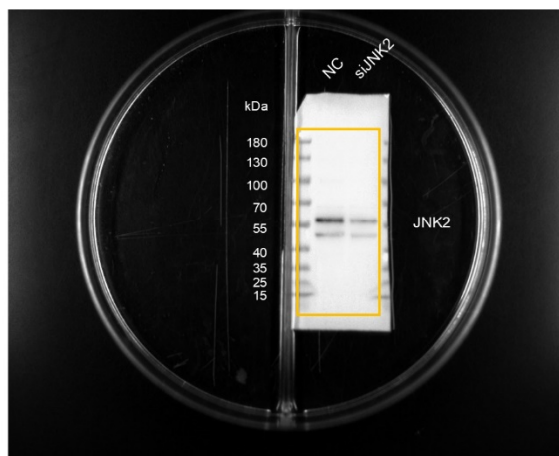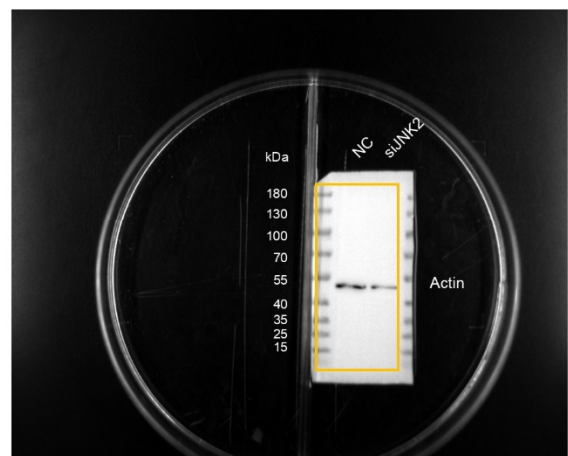

## siAURKA

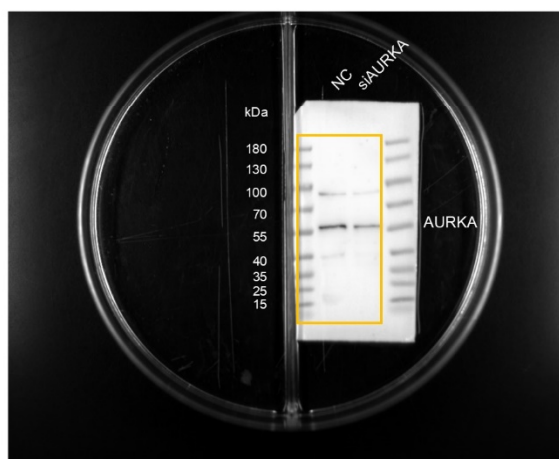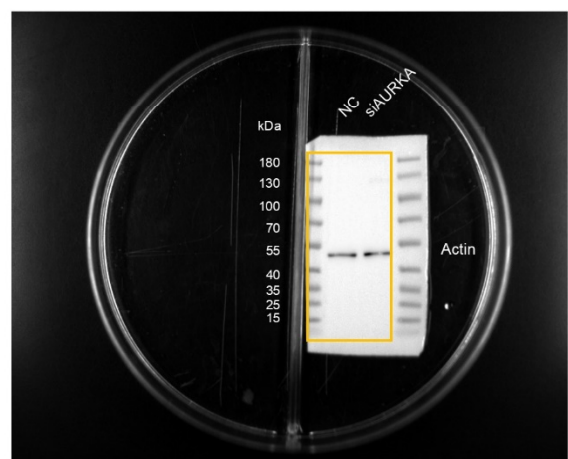

Supplement: SourceData FS5 — is the source file for Fig. S5. [file jcb_202503083_sourcedatafs5.pdf]
